# Can Self-reported Ocular Motor and Perceptive Alterations Predict a Reading Disability? A Pilot Study on the Analytic Anamnestic Protocol

**DOI:** 10.7759/cureus.5555

**Published:** 2019-09-02

**Authors:** Carlo Aleci

**Affiliations:** 1 Ophthalmology / Neuro-Ophthalmology, University of Turin, Turin, ITA

**Keywords:** self-report, dyslexia, perception, reading disability, sensibility, specificity, visuomotor, receiver operating characteristic (roc) curve, analytic anamnestic protocol (aap), polyunsaturated fatty acids (pufa)

## Abstract

Introduction

Early diagnosis is the main requisite when dealing with subjects suspected to suffer from neurodevelopmental disorders, especially reading disability. In this respect, self-reports are a promising tool and could prove to be as reliable as ordinary screenings, with the advantage of low cost and low time consumption. Since the last decades, the perceptual and visuomotor function are believed to be involved in the pathogenesis of developmental dyslexia; therefore, specific elements related to an alteration of the sensorial and visuomotor domain in the familial and personal medical history could reveal a risk to develop this condition at a pre-examination phase. Yet, rather than evaluating the perceptual and motor function, the self-reports presented so far investigate the presence of dyslexia traits and comorbidities in parents, relatives, and in the sons. The Analytic Anamnestic Protocol (AAP), specifically devised to assess the perceptual and visuomotor function in children, revealed higher visuomotor and sensorial scores in samples suffering from congenital cerebral lesions, Down syndrome, and reading disability compared to a control group. Sensibility and specificity were acceptable, as shown by the receiver operating characteristic (ROC) curves. In this paper, a modified version of the AAP (the AAP2) targeting more specifically developmental dyslexics is presented, along with the preliminary results obtained in a group of school-age disabled readers.

Methods

The AAP2 is made of 25 questions divided into four sections (family, general, past and recent specialist medical history). In addition, seven questions inquiring about aspects related to the lexical difficulties (developmental dyslexia (DD) section) have been included. Like the previous version, each answer is assigned a perceptive and visuomotor score. The self-report was administered to 37 normal subjects (median age: nine years), and 34 dyslexic children (median age: eight and a half years).

Results

Visuomotor and sensory scores in the dyslexic sample was consistently higher than in the controls in the recent specialist medical history and in the DD section (Welch test: visuomotor (VM) t = 7.02, p < .0001; sensory (VS) t = 7.39, p < .0001) with the visuosensory domain more involved than the visuomotor function (T-test: t = 4.70, p < .0001, and t = 7.06, p < .0001, respectively). The sensibility and specificity of the recent specialist medical history of the AAP was 94.12% and 77.78%, respectively. Sensibility and specificity of the DD section DD were 100% and 80%, respectively.

Conclusion

The AAP2 is a promising tool to screen subjects at risk for developmental dyslexia at the beginning of primary school. Like the previous version (also in this modified questionnaire), the main weakness remains the heuristic criterion adopted for the assignment of the scores.

## Introduction

Collecting precise and accurate medical history is the first fundamental step when it comes to diagnosing and managing a clinical condition. Its role is pivotal in the neuro-ophthalmological field when the symptoms depend not only on the type and severity of the disease but also on the way and on the extent it affects the visuomotor (M) and/or the perceptive (P) domain. In the last few years, a strand of research has managed to turn the medical history from a mere description of the clinical inheritance and past and recent pathological events into a quantifiable estimate that a given clinical condition is effectively occurring or will take place in the patient. To achieve this goal, most of these procedures turn the pieces of information that make up the familial and medical history into a numerical score, so that the higher the score, the higher the likelihood or the risk that the pathological event does occur.

Tosetto et al., for example, devised a score (they named it DASH) based on D-dimer, age, sex, and hormonal therapy to predict in subjects with unprovoked venous thromboembolism the recurrence of the disease [1]. More recently, Menekse et al. developed a score to predict mortality in patients with a perforated peptic ulcer [2]. Another example is the Framingham Risk Score, a specific algorithm that estimates the 10-year risk of coronary heart disease by scoring the most representative anamnestic informations, namely: age, sex, cigarette smoke exposure, total cholesterol, low-density lipoprotein (LDL) cholesterol, high-density lipoprotein (HDL) cholesterol, and systolic blood pressure [3].

As mentioned, score systems have been developed not only for prognostic purposes but also to improve diagnostic confidence. This is the case of the diagnostic score of Kruis, which weighs symptoms, such as pain and flatulence, laboratory findings like white blood cells count and erythrocyte sedimentation rate, and a history of blood in the stool, to maximize the probability of correctly diagnosing irritable bowel syndrome [4].

In the ophthalmological field, similar algorithms like the STAR (Scoring Tool for Assessing Risk) scoring systems [5] and the East London Glaucoma Prediction Score (ELGPS), have been introduced to judge the risk of developing glaucoma [6]. 

Early diagnosis, indeed, is a main requisite when dealing with subjects suspected to suffer from reading disabilities. It has been stated, in fact, that early diagnosis is essential to maximize the rehabilitative outcome in dyslexic children [7]. Yet, in this respect, two basic problems arise: first, since the diagnosis is based on the reading performance and since the rate of development of the lexical function differ significantly among children, the diagnosis of dyslexia is delayed until the third school grade; second, large scale screenings are time-consuming and, as for Italy, financially difficult to afford.

Self-reports could overcome these problems if they proved to be as reliable as ordinary screenings. As a matter of fact, a correlation between self-report and psychometric testing has been demonstrated in studies involving adult dyslexics, as well as parents of dyslexic children [8-14]. For example, Decker et al. found a correspondence between low scores at the self-report for reading disability and reading difficulty and consistent differences in the composite reading/spelling scores obtained from parents of dyslexic children compared to those of normal readers [9]. Schulte-Körne et al. showed that self-report data on spelling and reading difficulty satisfactory predict the spelling and reading disability diagnosed at the psychometric tests in parents of dyslexic children, with sensitivity ranging from 81% to 91% and specificity 84% - 88% [10]. The scores of the Adult Reading History Questionnaire (ARHQ), a self-report devised by Lefly and Pennington to assess reading disability in adults, showed good correlation with the estimates of reading performance (r: 0.57-0.70), with a sensitivity of 81.8% and a specificity of 77.5% [11] or of 84.5% and 83.3% [13]. The same applies to the Adult Reading Questionnaire (ARQ) formulated by Snowling et al. [12]. Interestingly, Tamboer et al. found that the predictive validity of their self-report questionnaire was higher than the predictive validity of the Multiple Diagnostic Digital Dyslexia Test for Adults (97% vs 90%) [14].

These studies are devoted to adult readers and substantially investigate the presence of dyslexia traits or comorbidities, providing a measure of literacy in the parents. This is achieved by asking subjects to judge each item of the questionnaire according to a grading scale so that higher scores reflect greater difficulties.

In the last few decades, a bulk of research (see Aleci [15] for a comprehensive review) supports the importance of visual impairment in the pathogenesis of developmental dyslexia. Epidemiological studies have established that dyslexic children do not suffer from particular visual problems during a standard clinical examination [16-19]. Yet, sensorial and visuomotor alterations in parents or in relatives in their own medical history could make them more susceptible to develop this condition.

It would, therefore, be advisable at a preliminary (i.e., preclinical examination) phase to identify and quantify the presence of a visuomotor and/or sensorial impairment in the familial and medical history of children admitted to a neuro-ophthalmological department.

For this purpose, the Analytic Anamnestic Protocol (AAP) has been developed. The AAP is made of multiple-choice questions divided into four sections (familial medical history (FH), general medical history (GH), past and recent specialist medical history (PSH, RSH)) aimed at collecting signs and symptoms reported by the patients or by their closest family members. At each question, visuomotor (VM) and sensory (VS) scores are assigned based on the choice of the subject [20]; the VM and VS cumulative score of the first section quantifies how strong familial risk factors may affect the visuomotor and sensory performance of the subject. Similarly, the VM and VS cumulative score of the remaining three sections estimate the likelihood that the general or the ophthalmological clinical conditions will affect the visuomotor and visuosensory performance of the child. By plotting the total VM and VS scores on a Cartesian graph, the overall extent and the relative proportion of visuomotor and visuosensory impairment can be represented.

In a previous study, the AAP was administered to a sample of children suffering from congenital cerebral lesions, Down syndrome, and reading disability [20]. Compared to a control sample, the three pathological groups showed higher scores, and the AAP was shown to be sensible and specific enough to orient the diagnosis of children at a preclinical examination phase.

To better investigate the usefulness of this approach for the clinical management of reading disabilities, a modified version of the AAP (the AAP2) has been tested in a sample of dyslexic pupils.

## Materials and methods

The AAP2

The original version (AAP) is a set of 27 questions organized into the four above-mentioned sections [20]. A number of possible answers are referred to each question, and parents have to mark which choice is the most suitable for their child. In the modified version used in this study (AAP2), there were 25 questions: one investigating the familial medical history, eight related to the general medical history, four related to the past specialist medical history, and 12 referred to the recent specialist medical history. In addition, seven questions (of which three were selected from the recent specialist medical history and focused on the characteristics proper of dyslexic subjects and four formulated to investigate signs of polyunsaturated fatty acids (PUFA) and especially omega 3 fatty acid deficiency) were included (“developmental dyslexia section,” DD section). As a matter of fact, omega 3 fatty acid deficiency seems a distinctive trait of disabled readers [21-24].

Like the previous version, each answer was assigned a perceptive and visuomotor score, whose value depended on the supposed relationship between the self-reported information and the visuomotor or sensory domain. At the end of each section, the final VM and VS scores were computed as the sum of the M and S score collected for each question and reflected the potential visuomotor and sensorial involvement. By plotting the total VM and VS scores, the degree of visuomotor and perceptive impairment expected in the patient can be represented. A copy of the questionnaire has been included in the Appendix.

Participants

Thirty-seven normal subjects (median age: nine years, interquartile range (IR): two years), and 34 dyslexic children (median age: eight and a half years, IR: one year) were recruited from the outpatient clinic of the Neuro-Opthalmology service at the University of Turin. The diagnosis of developmental dyslexia was provided by the reference neuropsychiatric service. Informed consent was obtained after the explanation of nature and the aim of the research, then the self-report was handed to the parents. Data were analyzed after all the questionnaires had been given back to the experimenter.

This study was approved by the University of Turin, School of Medicine as the topic of a bachelor dissertation presented on November 9, 2018 and was performed in accordance with the tenets of the declaration of Helsinki. All applicable institutional and governmental regulations concerning the ethical use of human volunteers were followed.

## Results

The average reading rate was 3.23 Syl/sec (± 0.94) in the control group, and 1.56 Syl/sec (± 0.65) in the dyslexic sample (t-test: p = < .0001).

Thirteen visuomotor or visuosensory scores were identified as outliers at Grubb’s test and, therefore, removed. 

Table 1 shows the VM and VS average scores in the two groups computed for each section of the AAP2.

**Table 1:** VM and VS Mean Scores (+/- SD) in the Two Groups Computed for Each Section of the Analytic Anamnestic Protocol (AAP2) DD: developmental dyslexia (DD section); FH: familial medical history; GH: general medical history; PSH: past specialist medical history; RSH: recent specialist medical history; SD: standard deviation; VM: visuomotor domain; VS: sensory domain

	CONTROLS		DYSLEXICS	
	VM	VS	VM	VS
FH	0	0.05 (± 0.33)	0	0.23 (± 0.60)
GH	0.41 (± 0.84)	0.41 (± 0.84)	0.32 (± 0.87)	0.32 (± 0.87)
PSH	0.72 (± 2.09)	0.36 (± 1.62)	0.18 (± 1.04)	0
RSH	5.9 (± 7.22)	7.59 (± 9.49)	17.47 (± 6.54)	20.78 (± 4.96)
TOTAL	8.37(± 9.56)	8.81 (± 9.49)	17.57 (± 6.50)	22.17 (± 6.47)
DD	4.50 (± 7.81)	6.54 (± 11.18 )	18.41 (± 6.85)	23.05 (± 5.74)

The average VM and VS scores computed in the FH, GH, and PSH were negligible and did not differ in the two groups. On the contrary, VM and VS scores in the dyslexic sample were consistently higher than in controls in the RSH section (Welch test: VM: t= 7.02, p < .0001; VS: t = 7.39, p < .0001).

When considering the outcome of the DD section, the average score in the dyslexic sample was about four times higher than in the controls (Welch test: VM: t = 7.92, p < .0001; VS: t = 7.91, p < .0001).

In the dyslexic sample, the visuosensory domain was more involved in the RSH section and in the DD section, as the VS score was higher than the VM score in both cases (t-test: t = 4.70, p < .0001, and t = 7.06, p < .0001, respectively).

In the normal group, no significant difference between VM and VS scores was found in the RSH section, whereas in the DD section, VS was higher than VM (t-test: t = 1.75, p = .08, and t = 2.58, p = .014, respectively).

**Figure 1:**
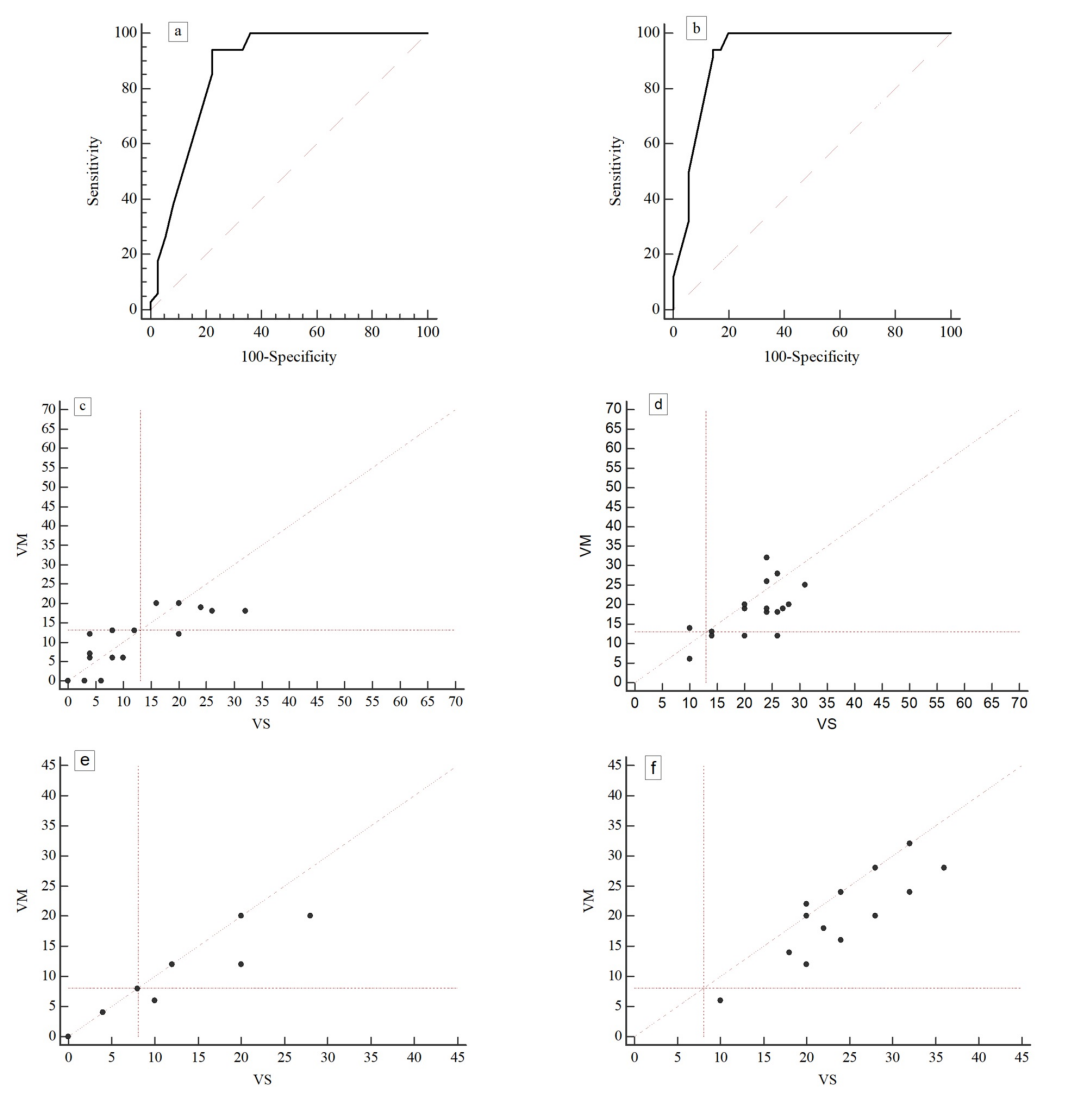
Receiver operating characteristic (ROC) curve and the distribution of the control and dyslexic observations Upper panels: ROC curve computed on the RSH section (a) and in the DD section (b) Middle panels: distribution of VM-VS scores computed in the RSH section of the questionnaire in the control group (c) and in the dyslexic sample (d) Bottom panels: distribution of VM - VS scores computed in the DD section: (e) control sample; (f) dyslexic sample. The perpendicular lines mark the best cutoff according to the Younden index. In the graphs, the observations appear less than expected due to overlapping DD: developmental dyslexia; RSH: recent specialist medical history; VM: visuomotor; VS: visuosensory

In each subject, the highest between VM and VS score in the RSH section was selected to plot a receiver operating characteristic (ROC) curve. AROC was 0.87, Younden index was 0.71 with associated criterion = 13. Therefore, setting as optimal cutoff a VM or VS score = 13, the sensibility and specificity of the RSH was 94.12% and 77.78%, respectively (Figure 1A).

The same procedure has been applied to the DD section. In this case, AROC was 0.92 and Younden index 0.8 with associated criterion < 8. Setting this score as optimal cutoff the sensibility and specificity of the DD was 100% and 80%, respectively (Figure 1B). Negative predictive value (computed on the prevalence of dyslexia in Italy of about 4%) was 100%, and the positive predictive value was 17.2%.

As shown in Figure 1C, the observations of the RSH section in the normal group are more concentrated to the lower left side of the graph and roughly displaced on both sides of the bisecting line. This suggests that normal subjects show scarce anamnestic positivity, involving the VM and VS domain to an overall equal degree. In turn, the observations of the RSH and DD section in the dyslexic group (Figure 1D, 1F) extend toward the right and tend to be localized below the bisecting line, therefore, showing preferential involvement of the visuoperceptive domain. 

## Discussion

The AAP is a computational procedure aimed at orienting on the clinical problem based on the medical history of patients suffering or supposed to suffer from ophthalmological and neuro-ophthalmological diseases. Its main goal is to provide the early identification of cases at risk in those clinical conditions where early diagnosis is particularly important for rehabilitative purposes. One of these conditions is developmental dyslexia. Great effort has been paid in the last years to identify children at risk for reading disability as soon as the kindergarten age. In this respect, the AAP2 is a useful tool, as it is able to quantify how well predisposing factors found in close relatives and the actual visual and visuomotor problems of the children may predict the onset of reading disabilities before the diagnosis is made. Evidently, this information cannot be provided by the conventional collection of data in medical history. An additional advantage is that the AAP2 can be administered as a screening tool not only by specialized physicians but also by optometrists or general practitioners.

In this pilot study, the AAP2 shows sufficient sensitivity and specificity when data obtained from the recent specialist medical history and from the DD section are considered.

In line with previous epidemiological studies [16-19], the negativity of familial, general, and past specialist medical history confirms that in a broad sense ophthalmological diseases are neither predisposing factors nor causally related to the lexical problems of disabled readers. In turn, the higher prevalence of recent and actual specific visual/visuomotor problems in the dyslexic sample suggests that (taken together) some of the symptoms and signs investigated by the questionnaire may predict the onset of a reading disability well before the third school grade (which is the age at which the diagnosis of dyslexia is generally made). Of the signs and symptoms, those involving the visuoperceptive domain seem more predictive than those related to the visuomotor function. This finding indirectly supports the theory that abnormal perception rather than non-optimal oculomotor function plays a role in dyslexia.

In a previous study, a former version of the AAP was administered to a sample of children suffering from congenital cerebral lesions, Down syndrome, and reading disability. Compared to a control group, the three pathological groups showed higher scores. Interestingly, if (in the former two pathological groups) the VM and VS score was roughly the same, a prevalent sensory involvement in the dyslexic sample was observed [20]. The sensibility and specificity of this first version of the AAP were satisfying, with the sensibility of 92.9% and specificity of 86.6% in the dyslexic sample.

The predictive power of this second version of the AAP is comparable to that of the former version of the AAP, but the number of questions (thereby, the time required to complete the questionnaire) is consistently lower.

## Conclusions

In conclusion, the AAP2 is a promising tool to screen subjects at risk for developmental dyslexia at the beginning of primary school.

Like the previous version (also in this modified questionnaire), the main weakness remains the heuristic criterion adopted for the assignment of the score. In addition, evidently, the results obtained in this study need confirmation in far larger samples.

## Appendices

Neuro-Ophthalmological Analytic-Anamnestic Protocol (AAP), 2nd Ed.

Date_______________________________

Name (or ID)    ________    ________                     Age___________

Consecutive number ______________

Address_________________________________________________________________________

Note: questions are formulated as if they were asked to the patient.

Familial Medical History

1. Parents or siblings who suffer or have suffered from eye diseases?

No

Yes, including:____________________________________________

1m    oculomotor palsy or strabismus

1s      glaucoma

1s      maculopathy

1s      optic neuropathy

2s      keratoconus

2s      amblyopia

3m    nystagmus  

General Medical History

2. Were you born prematurely?

No

1s+1m   Yes

3. Any problems at birth?

No

2s+2m Yes

4. Do you suffer from diabetes?

No

2s+1m    Yes, but I do not take any hypoglycaemic medicine; I keep blood sugar under control

through proper diet and as far as I am told I do not suffer from diabetic retinopathy

3s+2m    Yes,  but as far as I know I do not suffer from diabetic retinopathy

5s+3m    Yes, and I suffer from diabetic retinopathy

7s+5m    Yes, and I suffer from diabetic retinopathy, and I have undergone at least one laser treatment for diabetic retinopathy

5. Do you take medicines orally or parenterally?

No

Yes, including:_______________________________________________

     1s antiepileptics

     1s+1m anti-aggregates/anticoagulants

     1s chemotherapeutic agents

     3s hydroxychloroquine

 6. Do you suffer from neuro-ophthalmological diseases?

No

Yes, including:_____________________________________________

2s+5m Parkinson

5s+3m hydrocephalus

5s+1m occipital stroke

5s+3m temporal or parietal stroke

4m frontal stroke

4s+3m stroke (unspecified site of lesion)

7s+6m multiple sclerosis or other demyelinating diseases

7. Do you suffer from rheumatological diseases?

No

5s Yes (rheumatoid arthritis, lupus, scleroderma, others)

8. Do you suffer from Sjogren’s syndrome?

No

6s  Yes

9. Do you suffer from thyroidal diseases?

No

4s+4m   Yes

7s+7m   Yes, and I am taking thiamazole or thiamazole-like drugs

Past Specialist Medical History

10. Have you suffered in the past of ophthalmological diseases?

No

Yes, including: ______________________________________________     

4m   oculomotor palsy

6s    maculopathy

5s+ 8m   strabismus

6s    optic neuropathy

6s    keratoconus

7m   nystagmus  

2s    ocular trauma with past visual loss (now recovered)       

11. Have you ever undergone ocular surgery?

No

Yes, including:

8s intraocular injections for maculopathy

5m surgery for strabismus

5s refractive surgery

3s+2m retinal detachment surgery

12. Have you received in the past anti-amblyopic treatments (occlusion for lazy eye)?

No

Yes:

4s+4m lazy eye for strabismus

4s lazy eye not for strabismus

13. Were you administered in the past orthoptic exercises?

No

6m Yes

Recent Specialistic Medical History

14. Do you actually suffer from any diagnosed eye diseases?

No

Yes, including:_______________________________________________

12m oculomotor palsy

12s maculopathy

6m strabismus

12s optic neuropathy

12s keratoconus

8s amblyopia

8s+10m  nystagmus  

12s corneal diseases with  permanent visual loss

12s cataract

6s glaucoma

8s glaucoma  in therapy with 3 different eye drops, or that required surgery         

8m eyelid ptosis (the upper eyelid is droopy in the left or right eye, or both)

9m one or both eyelids look droopy in the evening compared to the morning

15. Are you having a chronic therapy with anti-histaminic drops or artificial tears?

No:

Yes:

3s with anti-histaminic drops

3s with artificial tears

16. Are you actually experiencing a satisfying vision (with or without glasses)?

Yes

8s No

6s Not always

17. Have you experienced transient visual blurring on some occasion?

No

Yes

8s at any time during the day

8s +6m especially in the evening

8s+7m when reading or using the pc

18. Do you frequently suffer from frontal headache in the evening (or more severe in the evening)?

No

4s+7m Yes

19. Do you frequently suffer from visual migraine?

No

3s Yes

20. Do you frequently have red eyes associated with a sense of itching or burning or stinging?

No

Yes

6s+6m at any time during  the day

4s+7m especially in the evening

6s+8m after reading or using the pc.

21. Do you ever see double?

No

Yes             

7m sometimes

9m especially at the evening

12m after reading or using the pc

22. Have you ever noticed sometimes you keep your head tilted?

No

4s+6m Yes

23. When reading don’t you ever mix up syllables?            

No

0s+6m Yes

24. When reading, do you ever see like jumping/moving letters?

No

8m Yes

25. When reading, do you ever reverse the syllables?       

No

10s+6m Yes

Section DD

1. When reading, do you ever mix up syllables?            

No

10s + 6m Yes

2. When reading, do you ever see jumping/moving letters?

No

8m Yes

3. When reading, do you ever reverse the syllables?

No

10s + 6m Yes

4. Do you have a dandruff problem?

No

8s+8m Yes

4s+4m Sometimes

5. Is your skin dry?       

No

8s+8m Yes

4s+4m Sometimes

6. Are you often thirsty?

Not particularly

4s+4m yes

7. Do you frequently feel the urge to urinate?      

Not usually

4s+4m  Yes

Data Elaboration

Visuosensory score (r) _______

Visuomotor score (v) _______

## References

[1] Tosetto A, Iorio A, Marcucci M (2012). Predicting disease recurrence in patients with previous unprovoked venous thromboembolism: a proposed prediction score (DASH). J Thromb Haemost.

[2] Menekse E, Kocer B, Topcu R, Olmez A, Tez M, Kayaalp C (2015). A practical scoring system to predict mortality in patients with perforated peptic ulcer. World J Emerg Surg.

[3] Lloyd-Jones D, Wilson P, Larson M, Beiser A, Leip E, D'Agostino R, Levy D (2004). Framingham risk score and prediction of lifetime risk for coronary heart disease. Am J Cardiol.

[4] Kruis W, Thieme CH, Weinzierl M, Schussler P, Holl J, Paulus W (1984). A diagnostic score for the irritable bowel syndrome: its value in the exclusion of organic disease. Gastroenterology.

[5] Walland M (2008). Use of the Medmont Automated Perimeter with the Scoring Tool for Assessing Risk (STAR) II glaucoma risk calculator. Clin Experiment Ophthalmol.

[6] Stephen C, Benjamin L (2013). The East London glaucoma prediction score: web-based validation of glaucoma risk screening tool. Int J Ophthalmol.

[7] Peterson RL, Pennington BF (2012). Developmental dyslexia. Lancet.

[8] Finucci JM, Whitehouse CC, Isaacs CC, Childs B (1984). Derivation and validation of a quantitative definition of specific reading disability for adults. Dev Med Child Neurol.

[9] Decker SN, Vogler GP, Defries JC (1989). Validity of self-reported reading disability by parents of reading-disabled and control children. Read Writ.

[10] Schulte-Körne G, Deimel W, Remschmidt H (1997). Can self-report data on deficits in reading and spelling predict spelling disability as defined by psychometric tests?. Read Writ.

[11] Lefly DL, Pennington BF (2000). Reliability and validity of the adult reading history questionnaire. J Learn Disabil.

[12] Snowling M, Dawes Piers, Nash H, Hulme C (2012). Validity of a protocol for adult self-report of dyslexia and related difficulties. Dyslexia.

[13] Bjornsdottir G, Halldorsson JG, Steinberg S, Hansdottir I, Kristjansson K, Stefansson H, Stefansson K (2014). The adult reading history questionnaire (ARHQ) in Icelandic: psychometric properties and factor structure. J Learn Disabil.

[14] Tamboer P, Vorst HCM, de Jong PF (2017). Six factors of adult dyslexia assessed by cognitive tests and self-report questions: Very high predictive validity. Res Dev Disabil.

[15] Aleci C (2013). Dyslexia: A Visual Approach. Nova Science Publishers Inc, New York.

[16] Norn MS, Rindziunski E, Skydsgaard H (1969). Ophthalmologic and orthoptic examinations of dyslectics. Acta Ophthalmol (Copenh).

[17] Aasved H (1987). Ophthalmological status of school children with dyslexia. Eye.

[18] Ygge J, Lennerstrand G, Axelsson I, Rydberg A (1993). Visual functions in a Swedish population of dyslexic and normally reading children. Acta Ophthalmol (Copenh).

[19] Ygge J, Lennerstrand G, Rydberg A, Wijecoon S, Pettersson BM (1993). Oculomotor functions in a Swedish population of dyslexic and normally reading children. Acta Ophthalmol (Copenh).

[20] Aleci C, Canavese L (2015). A computational approach to the anamnestic collection in neuro-ophthalmology: the Analytic Anamnestic Protocol. Research.

[21] Richardson A, Phil D (2001). Fatty acids in dyslexia, dyspraxia, ADHD and the autistic spectrum. Nutrition Pratictioner.

[22] Richardson AJ, Calvin CM, Clisby C (2000). Fatty acid deficiency signs predict the severity of reading and related difficulties in dyslexic children. Prostaglandins Leukot Essent Fatty Acids.

[23] Taylor KE, Higgins CJ, Calvin CM, Hall JA, Easton T, McDaid AM, Richardson AJ (2000). Dyslexia in adults is associated with clinical signs of fatty acid deficiency. Prostaglandins Leukot Essent Fatty Acids.

[24] Aleci C (2017). Rationale of polyunsaturated fatty acids supplementation in the frame of the magnocellular theory of dyslexia. J Adv Med Pharm Sci.

